# Molecular and pathological investigation of avian reovirus (ARV) in Egypt with the assessment of the genetic variability of field strains compared to vaccine strains

**DOI:** 10.3389/fmicb.2023.1156251

**Published:** 2023-04-17

**Authors:** Samah M. Mosad, Ehab Kotb Elmahallawy, Abeer M. Alghamdi, Fares El-Khayat, Manal F. El-Khadragy, Lobna A. Ali, Walied Abdo

**Affiliations:** ^1^Department of Virology, Faculty of Veterinary Medicine, Mansoura University, Mansoura, Egypt; ^2^Department of Zoonoses, Faculty of Veterinary Medicine, Sohag University, Sohag, Egypt; ^3^Department of Biology, Faculty of Science, Al-Baha University, Al-Baha, Saudi Arabia; ^4^Department of Poultry Diseases, Faculty of Veterinary Medicine, Kafrelsheikh University, Kafrelsheikh, Egypt; ^5^Department of Biology, College of Science, Princess Nourah bint Abdulrahman University, Riyadh, Saudi Arabia; ^6^Cell Biology and Histochemistry, Zoology Department, Faculty of Science, South Valley University, Qena, Egypt; ^7^Department of Pathology, Faculty of Veterinary Medicine, Kafrelsheikh University, Kafrelsheikh, Egypt

**Keywords:** avian orthoreovirus, ARV, sigma C, vaccine, phylogenetic analysis, histopathology

## Abstract

Avian orthoreovirus (ARV) is among the important viruses that cause drastic economic losses in the Egyptian poultry industry. Despite regular vaccination of breeder birds, a high prevalence of ARV infection in broilers has been noted in recent years. However, no reports have revealed the genetic and antigenic characteristics of Egyptian field ARV and vaccines used against it. Thus, this study was conducted to detect the molecular nature of emerging ARV strains in broiler chickens suffering from arthritis and tenosynovitis in comparison to vaccine strains. Synovial fluid samples (*n* = 400) were collected from 40 commercial broiler flocks in the Gharbia governorate, Egypt, and then pooled to obtain 40 samples, which were then used to screen ARV using reverse transcriptase polymerase chain reaction (RT-PCR) with the partial amplification of ARV sigma C gene. The obtained RT-PCR products were then sequenced, and their nucleotide and deduced amino acid sequences were analyzed together with other ARV field and vaccine strains from GenBank. RT-PCR successfully amplified the predicted 940 bp PCR products from all tested samples. The phylogenetic tree revealed that the analyzed ARV strains were clustered into six genotypic clusters and six protein clusters, with high antigenic diversity between the genotypic clusters. Surprisingly, our isolates were genetically different from vaccine strains, which aligned in genotypic cluster I/protein cluster I, while our strains were aligned in genotypic cluster V/protein cluster V. More importantly, our strains were highly divergent from vaccine strains used in Egypt, with 55.09–56.23% diversity. Sequence analysis using BioEdit software revealed high genetic and protein diversity between our isolates and vaccine strains (397/797 nucleotide substitutions and 148-149/265 amino acid substitutions). This high genetic diversity explains the vaccination failure and recurrent circulation of ARV in Egypt. The present data highlight the need to formulate a new effective vaccine from locally isolated ARV strains after a thorough screening of the molecular nature of circulating ARV in Egypt.

## 1. Introduction

Avian orthoreovirus is a member of the genus *Orthoreovirus*, which belongs to the *Spinareoviridae* family and Reovirales order, as classified by the International Committee on Taxonomy of Viruses^1^. With regard to its structure, ARV has a unique double-layered icosahedral capsid that measures about 70–80 nm in diameter without the envelope (Spandidos and Graham, 1976). The ARV particle has 10 double-stranded genomic RNA segments (1–4 kbp). The genomic segments are classified by polyacrylamide gel electrophoresis according to their size into three classes: small (S1–S4), medium (M1–M3), and large (L1–L3) (Spandidos and Graham, 1976). Segment S1 encodes three proteins, while each of the other nine segments translates into a single protein. S1 possesses three open reading frames (ORFs): ORF1 and ORF2 encode P10 and P17 non-structural proteins, respectively, whereas ORF3 encodes sigma C (δC) structural protein, which is located on the surface of the viral capsid and acts as a viral attachment protein and apoptosis inducer, selects specific neutralizing antibodies, and is considered the major antigenic determinant of ARV (Martínez-Costas et al., 1997; Shih et al., 2004; Benavente and Martínez-Costas, 2007). Based on the molecular characterization of sigma C protein sequences, six ARV genotypes have been identified (Kant et al., 2003; Ayalew et al., 2017; Palomino-Tapia et al., 2018). Most reports have stated that there are only six genotypic clusters of ARV isolated from chickens, while the seventh genotypic cluster is from wild birds (Kim et al., 2022). However, De la Torre et al. (2021) detected a new variant strain from poultry and classified ARV into seven genotypic clusters.

With regard to its evolution, the first report of avian reovirus being isolated from birds was in 1954 (Fahey and Crawley, 1954). Consequently, several ARV variants were isolated worldwide with high antigenic diversity. Most ARV infections are asymptomatic, and the virus can infect various avian species, causing a wide range of disease conditions, but broiler and broiler breeder chickens are the most clinically affected (Rosenberger et al., 1989; Jones, 2000; Davis et al., 2012). ARV is the principal cause of hock joint and footpad arthritis and tenosynovitis in young broiler chickens. The affected birds are usually unable to reach food and water, so their growth can be reduced and their production slow, and even death can occur with severe ARV infection (Gouvea and Schnitzer, 1982; Lee et al., 1992; Liu et al., 2003). Other disease conditions are also caused by ARV infection, including runting–stunting syndrome, malabsorption syndrome, hepatitis, myocarditis, pericarditis, enteritis, pneumonia, encephalitis, and immunosuppression (Robertson, 1986; Van der Heide, 2000). With regard to the impact, ARV infections in poultry cause extreme economic losses from increased mortality rates (up to 10%), low feed conversion rate, reduced weight gain, lack of performance, uneven growth rate, viral arthritis/tenosynovitis, diminished marketability of diseased birds, condemnation of affected carcasses, and secondary viral or bacterial infections (Van der Heide, 2000).

In Egypt, both live attenuated and inactivated ARV vaccines are available. Attenuated vaccines contain the S1133 or 2177 strain, while inactivated vaccines contain strains S1133, 1733, 2408, and SS412. Protection against reovirus mainly depends on the transfer of maternal antibodies through the yolk to the progeny, thus, breeding birds are vaccinated three to four times (Rekik and Silim, 1992; Van der Heide, 2000; Zhang et al., 2005). Despite the use of an integrated vaccination program for breeder birds in Egypt using both inactivated and modified live virus vaccines, offspring are not fully protected. ARV was first detected in Egypt in 1984 (Tantawi et al., 1984), then was identified serologically in several Egyptian governorates (Zaher and Mohamed, 2009; Abd El-Samie, 2014). It was then identified by RT-PCR from proventriculitis, tenosynovitis, and malabsorption syndromes (Kutkat et al., 2010; Ramzy et al., 2016; Mansour et al., 2018). Then, molecular characterization of ARV was performed based on the σA-encoding gene (Al-Ebshahy et al., 2020). It is important to mention that ARV has extreme inherent genetic variability because of the segmented RNA genome, which increases recombination and reassortment mutation events (Liu et al., 2003; Bányai et al., 2011).

The poultry industry worldwide has faced the consequences of ARV variant emergence since 2011 (Lu et al., 2015; Zhong et al., 2016; Gallardo, 2017; Sellers, 2017; Egaña-Labrin et al., 2019; Ayalew et al., 2020). The new variants have accompanied acute tenosynovitis, arthritis, and pericarditis even in broilers and breeders vaccinated against ARV (Van der Heide, 2000; Davis et al., 2012). As ARV is unaffected by certain disinfection methods and can survive for long periods in the environment, variants can be easily transferred between countries through imported chickens, processed chicken products, and eggs (Ayalew et al., 2020). In addition, because ARV is unaffected by pH, heat, certain disinfectants, and proteolytic enzymes, it is difficult to keep the virus away from chicken farms (Jones, 2000). Consequently, the best sustainable decision is to control ARV by using proper genetic and antigenic vaccines. Despite the high prevalence of ARV infection in Egypt, the genetic and antigenic nature of ARV is not clear, as no previous studies investigated the ARV sigma C gene in Egypt. Therefore, the current study aimed to detect the genotypic properties of emerging ARV strains in broiler chickens suffering from arthritis and tenosynovitis. To the authors' knowledge, this is the first study to carry out a molecular investigation of ARV based on the sigma C protein to assess genetic variability in comparison to the vaccine strains used in Egypt.

## 2. Materials and methods

### 2.1. Ethical considerations

This study was revised and approved by the Kafrelsheikh University Animal Care and Use Committee, Kafrelsheikh University, Egypt (code number KFS-2020/3).

### 2.2. Clinical samples

Samples (*n* = 400) were collected from 40 commercial broiler flocks located in Gharbia governorate, Egypt, during 2020–2021. Synovial fluid was collected from 10 diseased birds per farm and pooled, and this was considered one working sample. The diseased birds thought to be infected with ARV were 10–25 days old. For histopathological examination, samples from the heart, liver, spleen, and tendons, including synovial membranes, were freshly collected and fixed in formalin (10%). The studied flocks were not vaccinated against ARV, but their breeders were vaccinated at 6 weeks of age and again at 10 weeks of age using a modified live virus vaccine, then with inactivated reovirus vaccine at 17 weeks of age. Other pathogens causing arthritis and other systemic macroscopic and microscopic lesions as *Escherichia coli, Staphylococcus aureus*, and *Pseudomonas aeruginosa* were excluded from tested samples by multiplex PCR according to Ammara et al. (2014). Meanwhile, *Mycoplasma synoviae* and *Salmonella* sp. were also excluded by PCR as described elsewhere (Bencina et al., 2001; Shanmugasamy et al., 2011; Ammar et al., 2019).

### 2.3. Histopathological examination

Samples from the heart, liver, spleen, and tendons, including synovial membranes, were immersed in 10% buffered neutral formalin for fixation. Then, samples were subjected to routine tissue techniques, including dehydration, clearance, embedding, and casting. The paraffin blocks were cut *via* microtome into 4–5 μm sections, which were fixed on glass slides for further staining with hematoxylin and eosin.

### 2.4. Molecular identification of ARV

#### 2.4.1. Viral RNA extraction

Synovial fluid was used for the extraction of viral RNAs using a QIAamp^®^ Min Elut^®^ Virus Spin Kit (Qiagen, Hilden, Germany) according to the manufacturer's directions. The extracted RNA was then frozen at −80 °C until use in sigma C gene amplification.

#### 2.4.2. Reverse transcriptase polymerase chain reaction

A total of pooled 40 synovial fluids samples were tested for ARV by the RT-PCR partial amplification of the sigma C gene. RT-PCR was performed for the partial amplification of the ORF3 of genomic segment S1, which encodes ARV sigma C protein using a previously constructed primer set (Goldenberg et al., 2010). The oligonucleotide primer sequences were ARV δC F: 5′-TCMRTCRCAGCGAAGAGARGTCG-3′ and ARV δC R: 5′-TCRRTGCCSGTACGCAMGG-3′. These primers were synthesized by Metabion International AG (Steinkirchen, Germany). Qiagen one-step RT-PCR kit (Qiagen, Hilden, Germany) was used for RT-PCR according to the manufacturer's instructions. A Thermal cycler (T Gradient, Biometra, Germany) was set at an individual cycle of two steps: 50°C for 30 min and 94°C for 2 min; followed by 35 triple-step cycles: 94°C for 1 min, 50°C for 1 min, and 72°C for 1 min; with a single terminal elongation cycle of 72°C for 10 min. The 940 bp PCR products were then examined by agarose gel (1.5%) electrophoresis and an ultraviolet transilluminator (Biometra, Germany).

#### 2.4.3. Sequencing and phylogenetic analysis

The PCR products of positive samples with the strongest bands were selected, and their DNA was agarose gel purified with QIAquick PCR gel purification kits (Qiagen, Valencia, CA, USA), following the manufacturer's guidelines. The obtained DNA was submitted to Macrogen laboratory, South Korea, for forward and reverse sequencing. Direct nucleotide analysis was done according to several previous studies (Ayalew et al., 2017; Palomino-Tapia et al., 2018; Chen et al., 2019; Egaña-Labrin et al., 2019). The UniProt blast was also used for protein alignment to detect vaccine strain sigma C protein due to its divergence from our sequence. The protein sequences of the vaccines were also tracked and get their nucleotide sequence accession numbers then used in the NCBI nucleotide blast. The resulting sequences of the nucleotides of selected samples were then aligned using the BLAST tool in NCBI and then deposited in GenBank (http://www.ncbi.nlm.nih.gov/Genbank) with accession numbers OL741460 (Gharbia/1-20) and OL741461 (Gharbia/2-20). The sequences were then analyzed by ClustalW2 (https://www.ebi.ac.uk/Tools/msa/clustalw2/). The resulting files were analyzed to construct the phylogenetic tree with other reference sequences using MEGA X software (http://www.megasoftware.net/) for neighbor-joining phylogenic tree construction with a 1,000 repeat bootstrap test, p-distance substitution model, and pairwise deletion gap treatment together with other GenBank reference ARV sequences. BioEdit software version 7.1 was used for nucleotide and deduced amino acid sequence alignment using ClustalW (Hall, 1999).

## 3. Results

### 3.1. Clinical picture, postmortem lesions, and histopathological examination

**Figure 1:**
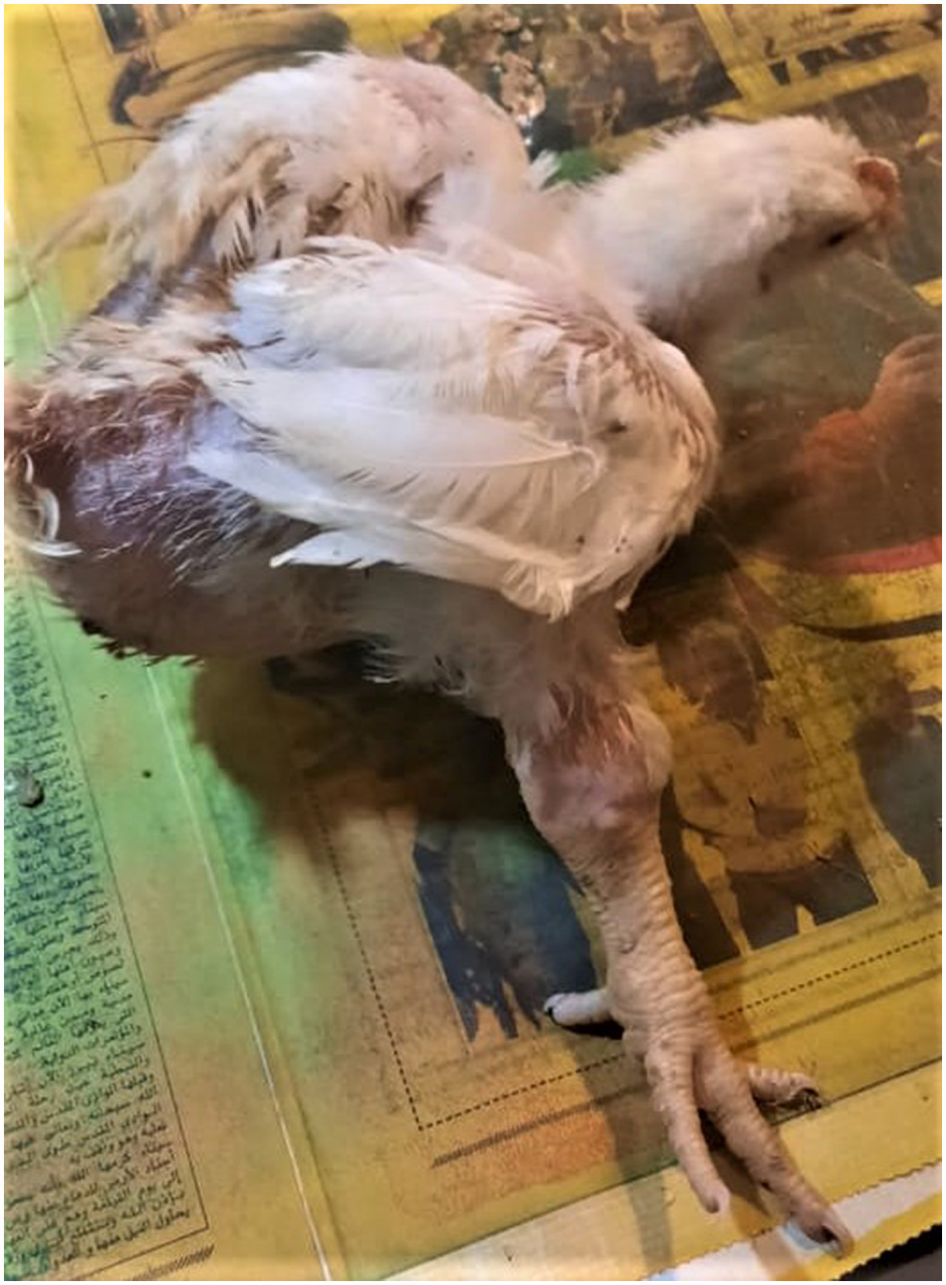
Clinical signs of ARV infections in broiler chickens. Broiler chicken with unilateral arthritis, hock joint swelling, and ruffled wing's feather.

**Figure 2:**
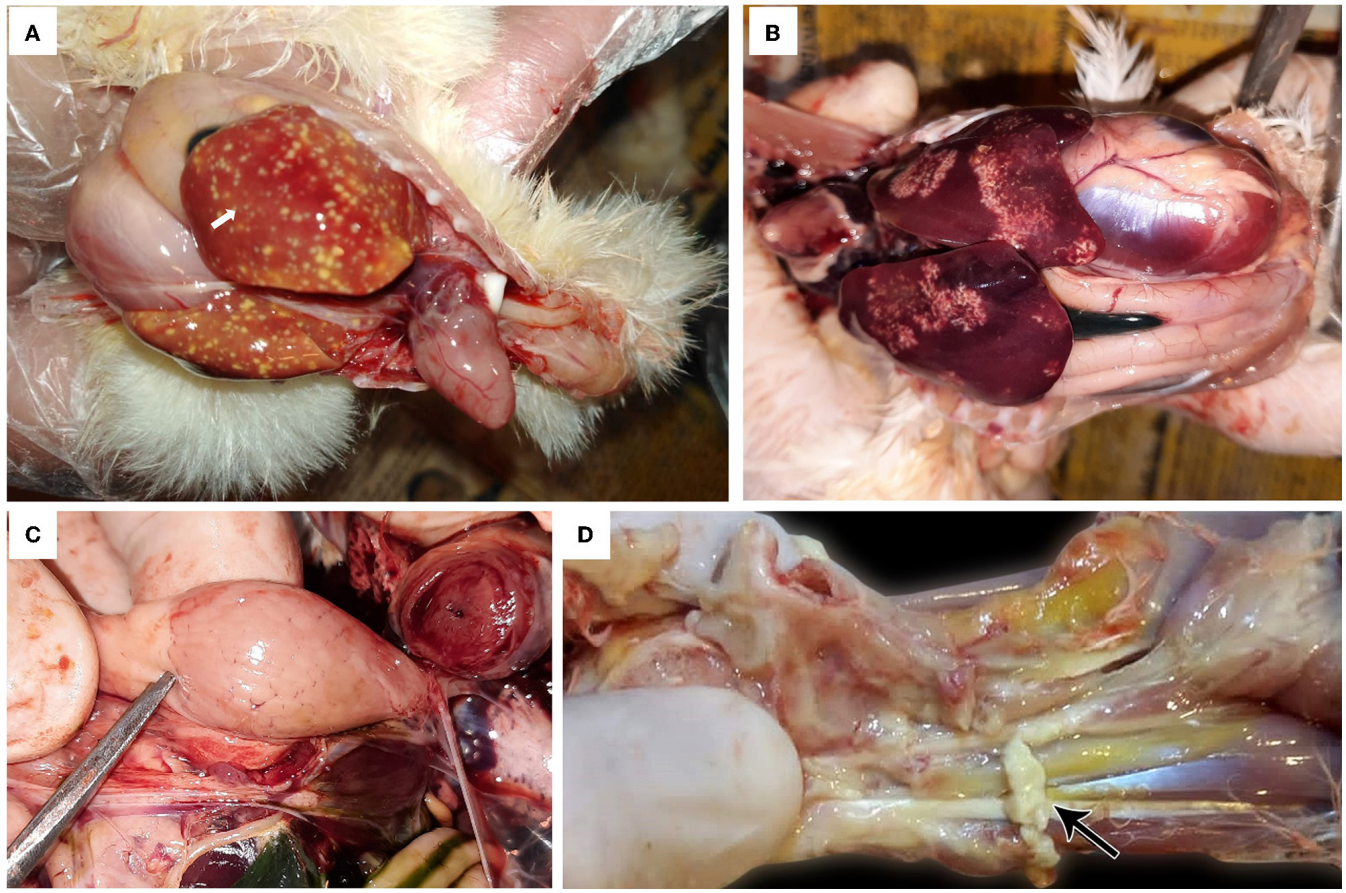
Avian orthoreovirus postmortem lesions in broiler chickens. **(A)** Broiler chicks show enlarged orange liver with numerous necrotic pale hepatic foci and hemorrhagic pericarditis (arrow). **(B)** Broiler chickens show hepatic necrosis. **(C)** Broiler chick with enlarged lemon-shaped proventriculus. **(D)** Broiler chicken with swelling, edema in the tendon, and full thickness tendon rupture (arrow).

The affected birds suffered from severe arthritis/tenosynovitis, which led to lameness and splay-leg with swelling of the hock joint and bumble foot (Figure 1). In some cases, the inflammation spread into the adjacent musculature. Uneven growth and growth retardation were also recorded, with 6% mortality and 50% culling rates. Upon the gross examination of affected carcasses, tendons and tendon sheaths showed marked edema, with tendon rupture (Figure 2D) and severe hemorrhage; pericarditis (Figure 2A), and myocarditis were also recorded in some cases. An enlarged lemon-shaped proventriculus (Figure 2C) with enlarged liver was reported, along with the orange discoloration of the hepatic parenchyma and miliary distribution of numerous pale tan necrotic hepatic foci (Figures 2A, B).

**Figure 3:**
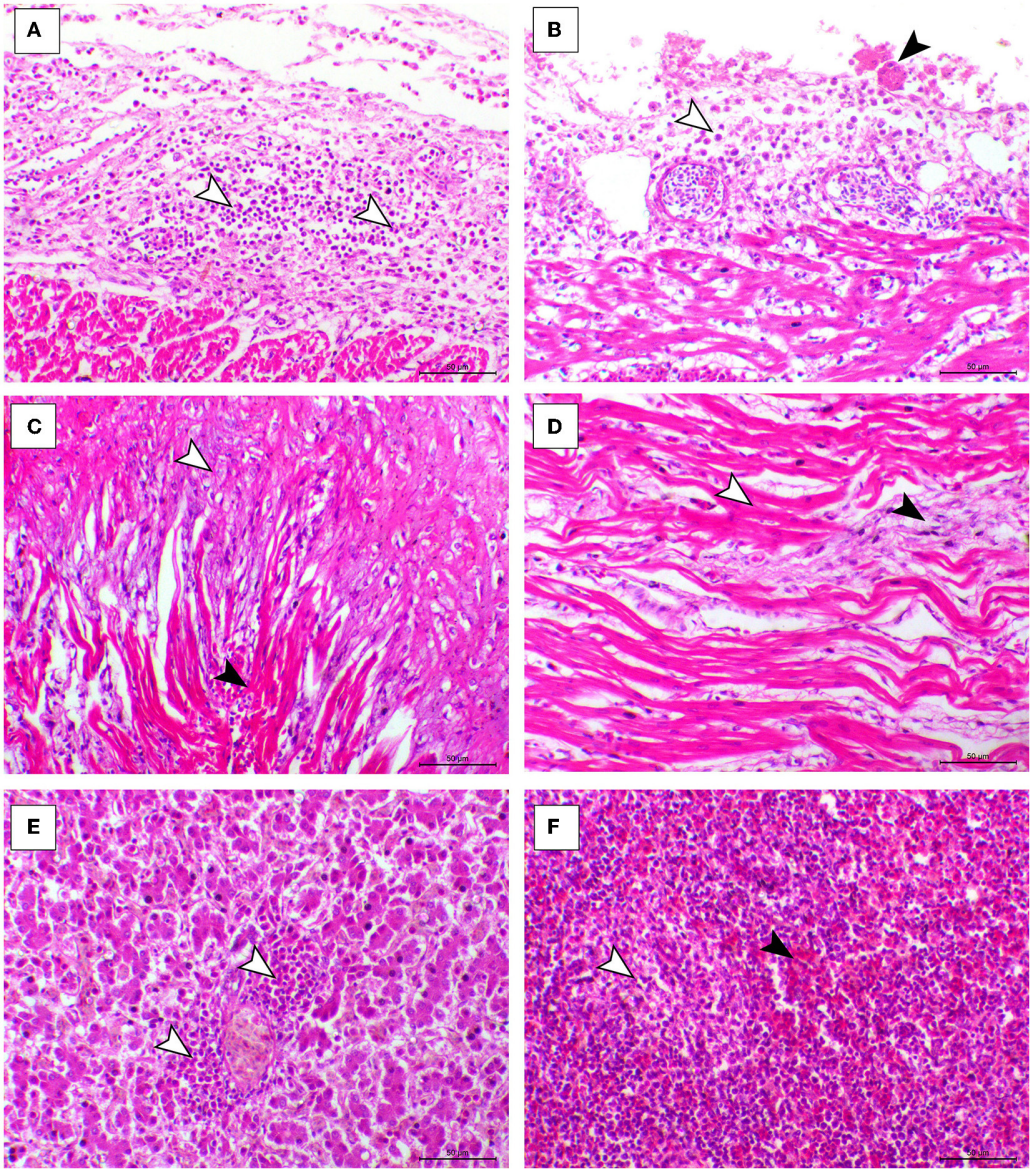
Cardiac, hepatic, and splenic histopathological lesions in the ARV-infected broiler chickens. **(A, B)** The pericardium of broiler chicks shows pericarditis associated with intense inflammatory cells infiltration (arrowheads). **(C)** Myocardium of broiler chicks shows marked atrophy of the myocardial cells (white arrowheads) and interstitial fibrosis (black arrowhead). **(D)** Myocardium of broiler chicks show myxomatous changes (white arrowhead) and severe eosinophilic sarcoplasmic degeneration (black arrowhead). **(F)** Liver of broiler chicks show periportal heterophilic infiltration (white arrowheads). **(E)** Liver of broiler chicks show the congestion of red pulp (black arrowhead) and moderate degree of lymphoid depletion (white arrowhead), H&E, bar = 50 μm.

**Figure 4:**
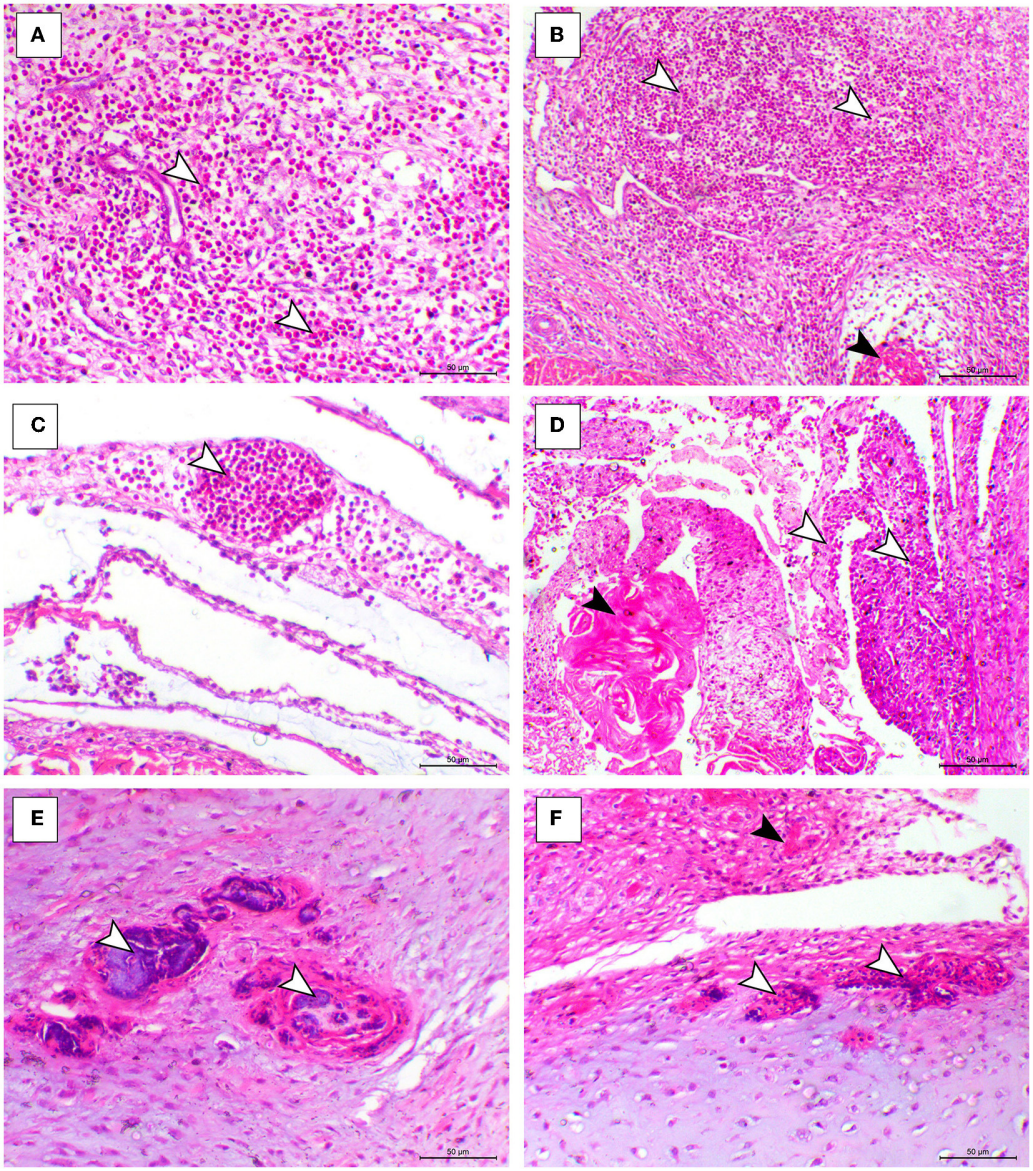
Synovial, tendon, and articular cartilaginous histopathological lesions in the ARV-infected broiler chickens. **(A–C)** Fibrin foci (black arrowhead) and marked heterophilic cell infiltration (white arrowheads) within the synovial membrane. **(D)** Necrosis o the synovial membrane (black arrowhead) and hypertrophy of synovial cells (white arrowhead). **(E, F)** Articular tissues showing multifocal necrosis on the surface or within the cartilage (white arrowhead), H&E, bar= 50 μm.

The histopathological features of infected birds are illustrated in Figures 3, 4. The examined heart sections showed pericarditis features extending to the myocardium. The pericardial sac showed massive fibrinous exudation with marked granulation tissue formation and capillary congestion, associated with severe inflammatory cell infiltration, including heterophils, lymphocytes, macrophages, and occasional syncytial cells. The myocardium revealed marked atrophy and degeneration rather than myocarditis. The inflammatory cells within the muscle layers were extended from pericardial lesions. There was marked interstitial cell proliferation. The endocardium showed severe degenerative changes ranging from sarcoplasmic eosinophilia to myxomatous changes. The liver of diseased birds showed marked sinusoidal congestion, hepatic degeneration, and periportal heterophilic cell infiltration. The spleen showed moderate to severe lymphoid depletion associated with histocytic cell infiltration. The synovial membranes showed severe synovitis, associated with necrosis of the synovial membranes admixed with fibrin within a granulation matrix and accompanied by severe heterophil infiltration. The tendons showed necrotic tendonitis associated with multifocal necrotic foci, with marked infiltration of inflammatory cells consisting of heterophils, lymphocytes, and macrophages. The articular surface showed multifocal necrotic changes in dystrophic calcification lesions.

### 3.2. RT-PCR, sequencing, and phylogenetic analysis

**Figure 5:**
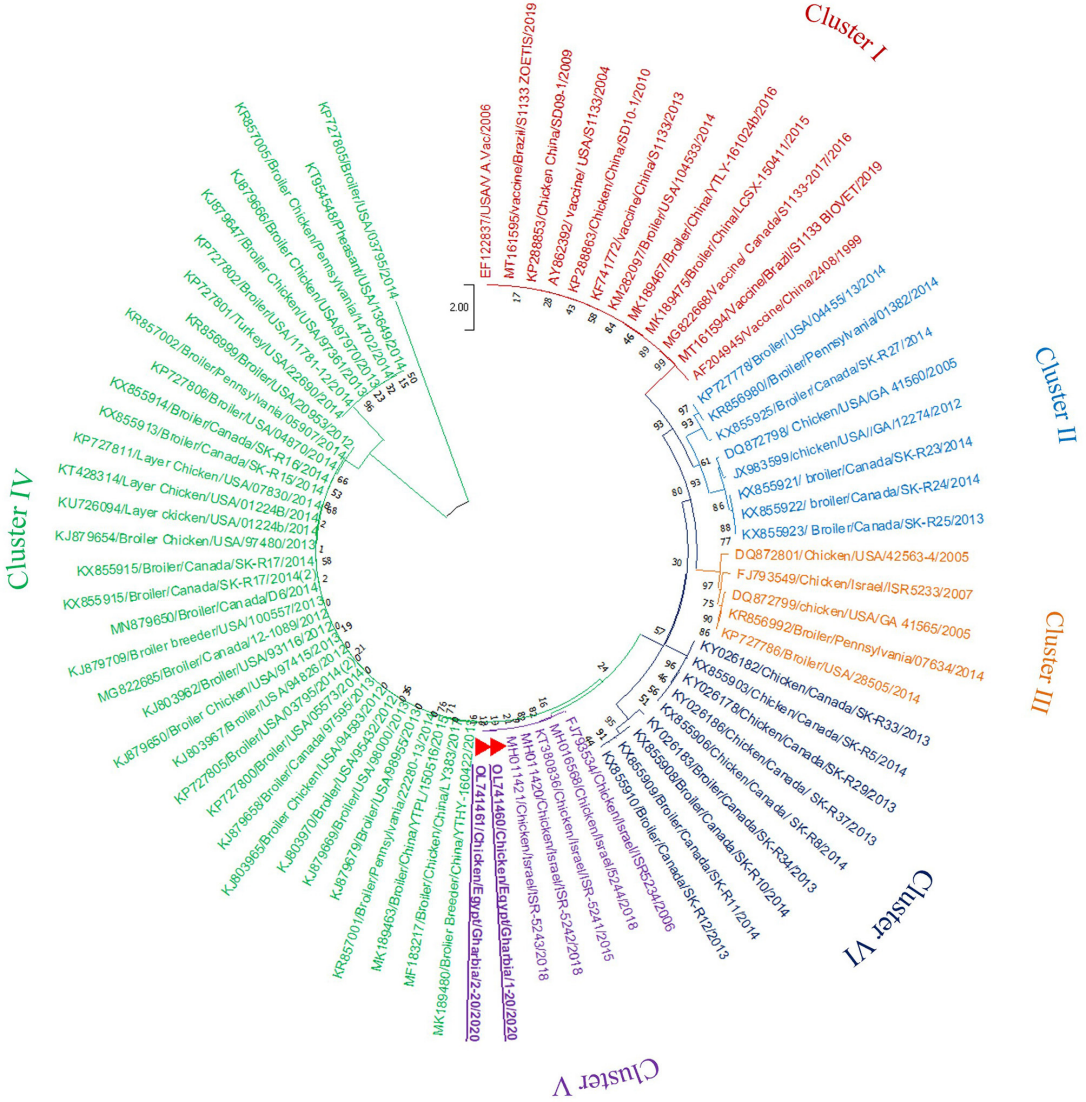
Phylogenetic tree of ARV sigma C gene nucleotide sequences constructed with MEGA X software in which the analyzed sequences are clustered into six genotyping clusters (genotypic cluster I–VI) according to Ayalew et al. (2017) ARV classification. Our isolates (red triangles) are clustered in genotypic cluster V away from all vaccine strains which are clustered in genotypic cluster I.

**Figure 6:**
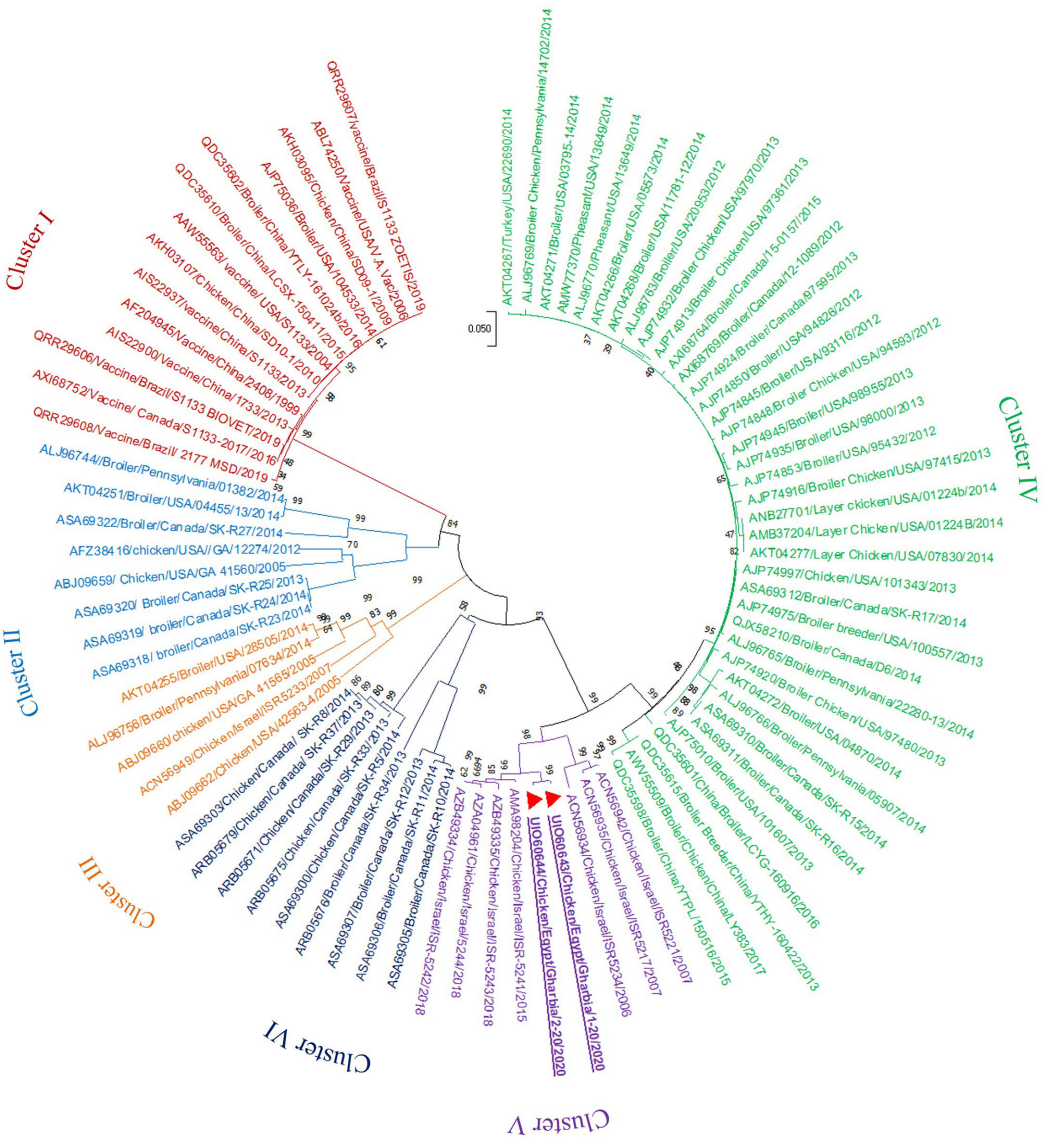
Phylogenetic tree of ARV sigma C gene deduced amino acid sequences constructed with MEGA X software in which the analyzed sequences are clustered into six antigenic clusters (antigenic cluster I–VI) according to Ayalew et al. (2017) ARV classification. Our isolates (red triangles) are clustered in the antigenic cluster V away from all vaccine strains which are clustered in the antigenic cluster I.

In the present study, RT-PCR successfully amplified the 940 bp fragment of the ARV sigma C gene from the 40 tested samples (100%). The Sigma C gene is usually used for genotyping and classifying ARV into protein clusters, as it is considered to be positioned in an extremely variable genomic region (Attoui, 2011; Kim et al., 2022). To study the genotypic properties of ARV, phylogenetic analysis of sigma C gene nucleotide sequences from our two selected isolates was performed together with 83 other sequences of reference field and vaccine strains retrieved from GenBank. Generally, the phylogenetic tree revealed that all 85 strains involved in the phylogenetic analysis were classified into six genotypic clusters (I–VI) (Figure 5). Based on ARV classification (Ayalew et al., 2017), all vaccine strains were clustered in cluster I. Surprisingly, our isolates were genetically different from vaccine strains and were clustered in genotypic cluster V with other isolates from Israel (the same geographic location). In addition, phylogenetic analysis of the deduced amino acid sequences from the sigma C gene of our two selected ARV isolates and the 83 reference strains from GenBank (the same strains used in nucleotide sequence analysis) was performed to study the deduced sigma C protein relatedness. All ARV strains were clustered into six clusters (I–VI) (Figure 6); the vaccine strains were in cluster I, while our isolates differed from those and were in cluster V with other isolates from Israel.

The diversity among the six deduced sigma C protein clusters was calculated with MEGA X software, which revealed high protein diversity among the six genotypic clusters. The lowest detected diversity was 13.21% between cluster V (ISR-5241) and cluster IV (LCYG-160916). Shockingly, the highest deduced protein diversity (57.65%) was between cluster V (including our strains) and cluster I (including the vaccine strains). The highest deduced protein diversity was between the ISR-5242 and S1133 vaccine strains. The diversity within clusters ranged from 0 to 39.62%; specifically, the ranges for clusters I–VI were 0.0–2.64%, 0.0–34.72%, 2.26–20.38%, 0.0–7.92%, 0.38–13.64%, and 0.0–39.62%, respectively (Supplementary Table 1). Surprisingly, our strains were highly divergent from vaccine strains used in Egypt: 55.47–56.23% between Gharbia/1-20 and vaccine strains, and 55.09–55.85% between Gharbia/2-20 and vaccine strains.

**Figure 7:**
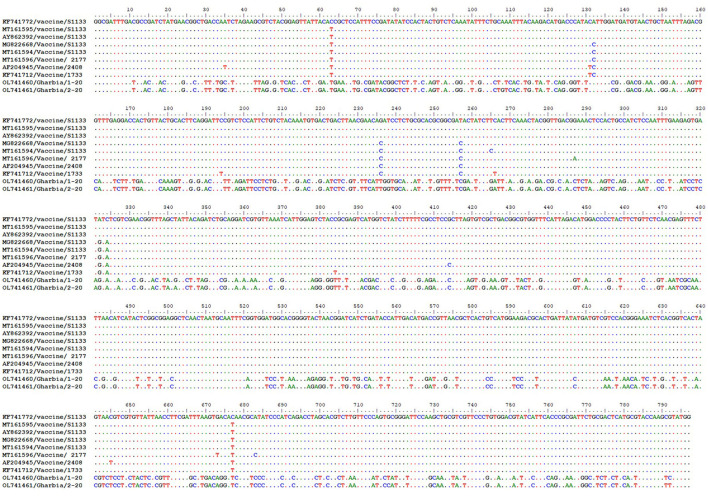
Avian orthoreovirus sigma C gene nucleotide sequences analysis using BioEdit software showing 397 nucleotide substitutions (out of 797 aligned nucleotides) in our isolates in comparison to vaccine strains used in Egypt.

**Figure 8:**
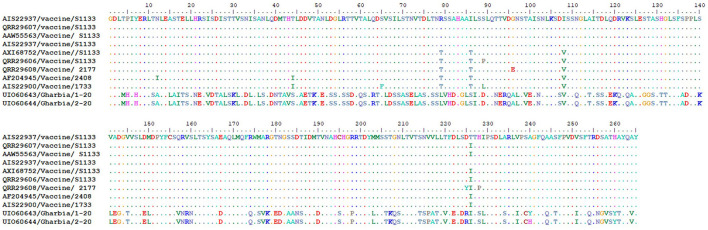
Avian orthoreovirus sigma C gene deduced amino acid sequences analysis using BioEdit software showing 149 (Gharbia/1-20) and 150 (Gharbia/2-20) amino acid substitutions (out of 265 aligned amino acids) in our isolates in comparison to vaccine strains used in Egypt.

To detect the genetic and deduced sigma C protein diversity between our two isolates and vaccine strains used in Egypt, the nucleotide sequences of the sigma C gene and its deduced amino acid sequences were analyzed with BioEdit software using vaccine strain S1133 (KF741772) as a reference. Nucleotide sequence analysis revealed high genetic diversity between our isolates and vaccine strains, with a substitution of 397 out of 797 aligned nucleotides (49.81%), as shown in Figure 7. Amino acid sequence analysis revealed high diversity between our isolates and vaccine strains, with a substitution of 149 (Gharbia/1-20) and 148 (Gharbia/2-20) out of 265 aligned amino acids (55.85 and 56.23%, respectively), as shown in Figure 8. On the other hand, there were few or no substitutions in nucleotide and amino acid sequences between vaccine strains and reference strain S1133 (Figures 7, 8). Our two isolates (Gharbia/1-20 and Gharbia/2-20) are genetically similar, with five nucleotide substitutions (A191G, G425A, A445G, T801C, and C875T) leading to two amino acid substitutions (Q148R and Y267H).

## 4. Discussion

Despite regular vaccination of breeder chickens against ARV in Egypt, pathogenic ARV infection remains a great challenge to the poultry industry, causing severe economic losses. The ineffectiveness of conventional vaccines and the recurrence of emergent pathogenic strains are alarming and threaten the broiler poultry industry. Moreover, ARV infection in broiler chickens at poultry farms that use uncommon ARV vaccinations has been increasingly observed in Egypt. As there are scarce studies on such an important disease in Egypt, genetic and antigenic characterization of emerging ARV strains is essential.

In the present study, 40 pooled synovial fluid samples were collected from 40 unvaccinated commercial broiler flocks, but their breeders were vaccinated (three doses of ARV vaccine). However, ARV is included in many pathological syndromes; the only major ARV-related clinical presentations are viral arthritis and runting–stunting syndrome (Jones and Guneratne, 1984; Rosenberger et al., 1989). Our samples were collected from birds suffering from arthritis/tenosynovitis and runting–stunting syndrome, which commonly affects broiler chickens infected with ARV (Robertson, 1986; Benavente and Martínez-Costas, 2007; Ayalew et al., 2017). In the present study, we observed bilateral arthritis in most studied chickens with marked swelling in both hock joints, while in previous studies (Jones and Guneratne, 1984) either unilateral or bilateral arthritis could be observed in ARV infection. The most characteristic clinical signs and postmortem evidence of runting–stunting syndrome observed in this study were stunted growth and enlarged lemon-shaped proventriculus with reduced gizzard size, as described elsewhere (Page et al., 1982; Hieronymus et al., 1983). In relation to microscopic lesions, as shown in our study, tenosynovitis, myocarditis, and hepatitis were the most common pathological lesions associated with ARV in the affected birds. The noticed lesions were consistent with previously reported data (Souza et al., 2018; Choi et al., 2022). Similarly, the experimental infection of ARV showed an interesting finding regarding the appearance of the ARV lesions, and footpad infection demonstrated earlier hock lesions mostly after 2 days of infection (Chen et al., 2015). In relation to systemic infection, it was accompanied by pericarditis, hepatitis, pancreatitis, and bursal atrophy. Lymphocytes and macrophages were the most components of the infiltrate. Taken into consideration, the bursa and intestinal mucosa may be a site for the initial replication of ARV, while the joints were the most serious consequence of viral replication (Jones, 2000). Importantly, the macrophages play a key role in viral replication and transmission (Mills and Wilcox, 1993). In the present study, the hepatic lesion showed multifocal yellowish foci, which is consistent with several previous reports (Choi et al., 2022). The foci consisted mostly of mononuclear cells consisting of lymphocytes and macrophages. In certain cases, lympho-histocytic infiltration and polykaryocytes appearance were reported (Mandelli et al., 1978). Interestingly, the main sites of the lesions as the hock joint, tendon, and lymphoid organs were the most organs used for viral isolation (Choi et al., 2022).

Along with the observed clinical signs, microscopic findings, and postmortem changes, the RT-PCR results confirmed the presence of ARV in all studied chickens, indicating that the arthritis was almost certainly ARV related. RT-PCR partial amplification of the sigma C gene was sensitive, and detected the virus in 100% of tested samples, while another study (Tang and Lu, 2016) concluded that some false-negative results could occur with sigma C gene amplification, as the sigma C sequence is highly variable. Even though the sigma C protein is within the most hypervariable region of ARV proteins (Liu et al., 2003), some other conserved sequences were identified within the sigma C protein (Goldenberg et al., 2010). Molecular characterization and genotypic correlation studies contribute to our understanding of the epidemiology, source, and evolution of emergent viral variants (Kant et al., 2003; Kort et al., 2015). Such studies mainly depend on the sequence of the extremely variable sequence in the ARV genome (sigma C gene), which is usually used to genotype ARV and classify it into antigenic clusters (Liu et al., 2003; Calvo et al., 2005; Goldenberg et al., 2010; Kort et al., 2015; Lu et al., 2015; Ayalew et al., 2017). As the efficacy of a vaccine mainly relies on its genetic and antigenic relatedness to field strains, an analysis of the genetic variability of Egyptian field ARV strains in comparison to vaccine strains depending on the sigma C protein sequence was performed to gain a better understanding of the antigenic variability among circulating ARV strains in Egypt.

Phylogenetic analysis of the sigma C gene nucleotide and deduced amino acid sequences from our two selected isolates was performed, together with sequences of reference field and vaccine strains retrieved from GenBank. Generally, the phylogenetic trees revealed that all strains included in the phylogenetic analysis were genetically diverse and clustered into six genotypic clusters and six sigma C protein-based clusters, as described elsewhere (Liu et al., 2003; Kort et al., 2015; Lu et al., 2015; Sellers, 2017; Palomino-Tapia et al., 2018). De la Torre et al. (2021) detected seven genotypic clusters from poultry, and Kim et al. (2022) characterized six genotypic clusters from poultry and an additional seventh genotypic cluster from wild birds. The genetic diversity between established ARV strains and new variant strains may be due to accumulated mutations and numerous reassortment actions (Ayalew et al., 2020). Surprisingly, all vaccine strains were clustered in genotype I and deduced protein cluster I, and our isolates were genetically different, clustered in genotype V and deduced protein cluster V with other isolates from Israel (the same geographic location), suggesting that the genotype V and deduced protein cluster V strains might have the same epidemiological origin. Several studies (Kant et al., 2003; Liu et al., 2003; Goldenberg et al., 2010) reported that it was not possible to relate ARV infection to geographic location, but another study (Ayalew et al., 2020) reported that there was widespread geographic intermixing of ARV, with the non-specific distribution of the six ARV genotypes in different countries around the world, which suggests that effective ARV prevention cannot be achieved without a vaccine formulation that contains the proper antigens from all ARV genotypes.

Our results revealed high genetic diversity among the six clusters (13.21 to 57.65%), similar to a previous report (Goldenberg et al., 2010) that recorded 50% diversity among studied isolates. Despite the regular vaccination of breeder chickens against ARV, there have been recurrent ARV outbreaks in different localities in Egypt, and the vaccines are based on strains isolated in the USA. The parent strains of these vaccines (S1133, 2177, 2035, 2408, and 1733 vaccine strains) were developed in the late 1970s and early 1980s (Van der Heide et al., 1983; Rosenberger et al., 1989). Therefore, the failure of ARV vaccination together with the increased incidence of ARV infections may be due to the newly emerging strains, which are genetically divergent from the vaccine strains. This may explain the observed vaccination failure associated with traditional vaccines, and it significantly increases the complication and difficulty of controlling and preventing ARV infections effectively. These suggestions were supported in previous studies (Ayalew et al., 2017). The vaccine strains have been shown to be ineffective at protecting against ARV infection because the RNA nature of this virus causes different mutation events, generating new variant strains that are incompletely neutralized by the antibodies produced against classical vaccine strains. Therefore, prompt characterization and genotyping of the strains causing disease in the field is needed, then autogenous vaccines based on the field strains can be formulated (Goldenberg et al., 2010; Lublin et al., 2011; Sellers et al., 2013; Troxler et al., 2013).

Our results revealed up to 39.62% diversity within the deduced sigma C protein clusters, while another study (Chen et al., 2019) recorded 45.7–51.4% amino acid diversity between their field isolate and vaccine strains. Surprisingly, our strains were highly divergent from vaccine strains used in Egypt, showing 55.09–56.23% diversity, which explains the vaccination failure and recurrent circulation of ARV. These results were confirmed by a study (Palomino-Tapia et al., 2018) that stated that an effective autogenous vaccine program would require at least 95% amino acid similarity between this vaccine strain and the circulating field strains. Some countries have begun to formulate autogenous ARV vaccines from local field strains, such as in Israel, where an autogenous inactivated ARV vaccine was formulated from local field strain 641 to achieve a better immune response (Goldenberg et al., 2010). The present study has some limitations which include the limited number of sequenced samples and the use of degenerated primer set targeting the sigma C protein coding ORF which is a high variable gene.

## 5. Conclusion

Our results suggest that ARV variants can evade the immunity of commonly used vaccine strains, as our field strains highly diverged from the vaccine strains used in Egypt. These findings reflect the necessity of formulating a new effective vaccine from locally isolated ARV strains, following a thorough screening of the molecular nature of circulating ARV strains in Egypt. These findings provide novel baseline information about the genetic information of the virus.

## Data availability statement

The original contributions presented in the study are included in the article/Supplementary material, further inquiries can be directed to the corresponding authors.

## Ethics statement

The animal study was reviewed and approved by the Ethics Committee of the Kafrelsheikh University Animal Care and Use Committee, Kafrelsheikh University, Egypt with code number; KFS-2020/3.

## Author contributions

SM, EE, AA, and WA were involved in the conception of the idea and methodology design and performed data analysis and interpretation. SM, EE, AA, FE-K, ME-K, LA, and WA participated in the methodology design, sampling and laboratory work, and data analysis. SM, EE, and WA contributed their scientific advice, prepared the manuscript for publication, and completed revisions. All authors have read and agreed to the published version of the manuscript.

## Footnotes

^1^ICTV. Available online at: https://ictv.global/taxonomy (accessed 9 September 2022).
